# Microencapsulation with alginate/CaCO_3_: A strategy for improved phage therapy

**DOI:** 10.1038/srep41441

**Published:** 2017-01-25

**Authors:** Joan Colom, Mary Cano-Sarabia, Jennifer Otero, Javier Aríñez-Soriano, Pilar Cortés, Daniel Maspoch, Montserrat Llagostera

**Affiliations:** 1 Departament de Genètica i Microbiologia, Universitat Autònoma de Barcelona, Campus de Bellaterra, Bellaterra, 08193 Cerdanyola del Vallès, Spain; 2 Catalan Institute of Nanoscience and Nanotechnology (ICN2), CSIC and the Barcelona Institute of Science and Technology, Campus UAB, Bellaterra, 08193 Cerdanyola del Vallès, Spain; 3 ICREA, Pg. Lluís Companys 23, 08010 Barcelona, Spain

## Abstract

Bacteriophages are promising therapeutic agents that can be applied to different stages of the commercial food chain. In this sense, bacteriophages can be orally administered to farm animals to protect them against intestinal pathogens. However, the low pH of the stomach, the activities of bile and intestinal tract enzymes limit the efficacy of the phages. This study demonstrates the utility of an alginate/CaCO_3_ encapsulation method suitable for bacteriophages with different morphologies and to yield encapsulation efficacies of ~100%. For the first time, a cocktail of three alginate/CaCO_3_-encapsulated bacteriophages was administered as oral therapy to commercial broilers infected with *Salmonella* under farm-like conditions. Encapsulation protects the bacteriophages against their destruction by the gastric juice. Phage release from capsules incubated in simulated intestinal fluid was also demonstrated, whereas encapsulation ensured sufficient intestinal retention of the phages. Moreover, the small size of the capsules (125–150 μm) enables their use in oral therapy and other applications in phage therapy. This study evidenced that a cocktail of the three alginate/CaCO_3_-encapsulated bacteriophages had a greater and more durable efficacy than a cocktail of the corresponding non-encapsulated phages in as therapy in broilers against *Salmonella*, one of the most common foodborne pathogen.

In farm animals and in aquaculture, antibiotics have been used not only in the treatment of infections but also as growth promoters. However, this intensive use of antibiotics has given rise to the emergence of resistant strains of commensal and pathogenic microorganisms that are able to spread to humans, either directly through the food chain or indirectly via the environmental pollution caused by farm effluents^1^. The European Food Safety Authority (EFSA) has played an essential role in monitoring and detecting the risks posed by the emergence of multi-drug resistant bacteria within the food industry^2^. As an alternative to the use of antibiotics, bacteriophages are promising therapeutic agents due to their ubiquity, high specificity, replication capacity inside their host, and their ease of isolation and production. The use of bacteriophage-based products in different stages of the food chain^3^,^4^ as well as in humans^5^,^6^ and animals^4^,^7^ via different routes of administration has been investigated. However, a challenge of oral phage therapy is that the phages must overcome numerous adverse conditions, including the low pH of the stomach, the activity of bile, and the activities of enzymes in the digestive tract^8^,^9^,^10^ and also their short residence time in the digestive tract^11^. In addition, when used in food protection bacteriophages may interact with food components and be exposed to unfavourable conditions or their stability during food processing and storage may be compromised. These problems can be successfully addressed by encapsulation, which would be able to protect bacteriophages under similar potentially deleterious conditions. Among the many diverse biomaterials used for phage encapsulation are cellulose, liposomes, alginate, whey proteins, and gelatin, which have been applied using different techniques^12^,^13^. Alginate is one of the most common biomaterials used in microencapsulation, including that of probiotics^14^,^15^ and, as recently shown, bacteriophages. The latter was achieved using alginate either alone or in combination with other components, in which wet capsules ranging in size from 310 μm to almost 1 mm were obtained^8^,^10^,^16^,^17^,^18^. Alginate, a linear copolymer containing blocks of (1, 4)-linked β-D-mannuronate and α-L-guluronate residues, is of low toxicity and immunogenicity and thus regarded as a biocompatible material^19^. When used as a hydrogel in biomedical applications^19^, alginate is combined with ionic cross-linking agents, such as divalent cations. In oral bacteriophage therapy, it protects the phage by shrinking at low pH values, dissolving at high pH values^8^, and participating in mucoadhesive interactions with gastrointestinal mucus^20^. These results were obtained from *in vitro* studies that examined the interactions of alginate-encapsulated bacteriophages with simulated gastric and intestinal fluids. By contrast, the use of alginate-encapsulated bacteriophages in oral phage therapy has been tested in only a few *in vivo* experiments involving *Salmonella* in pigs^21^,^22^. To our knowledge, the present study is the first in which wet bacteriophage-containing alginate/CaCO_3_ microcapsules <150 μm in size were obtained and then used as oral therapy to protect broiler chickens against *Salmonella* infection, under conditions mimicking those of poultry production farms. In particular, a cocktail composed by three virulent bacteriophages (UAB_Phi20, UAB_Phi78, and UAB_Phi87) was prepared according to previous promising results of phage-therapy studies^11^,^23^,^24^.

## Results

### Characterization of alginate/CaCO_3_-encapsulated phages

**Figure 1:**
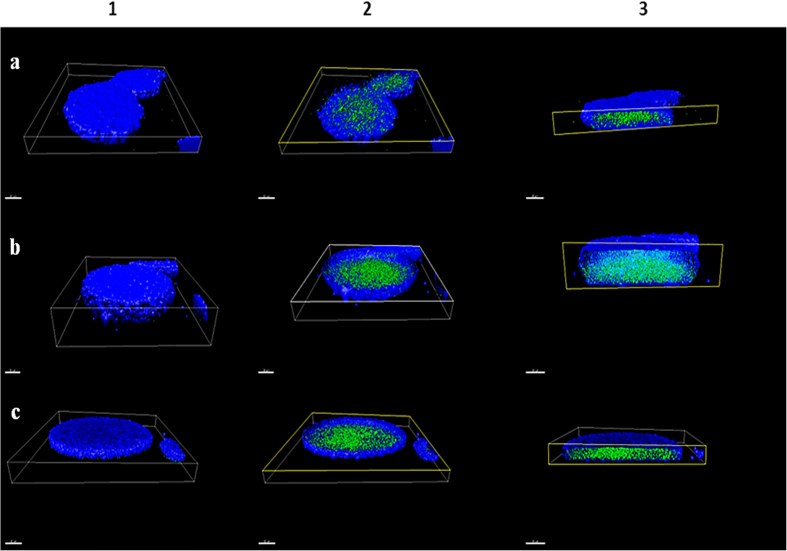
3D confocal microscopy images of SYBR-gold-labelled UAB_Phi20 (**a**), UAB_Phi78 (**b**), and UAB_Phi87 (**c**) (green) encapsulated in DAPI-labelled alginate capsules (blue). 3D images of the capsules are shown on the left (1), and the corresponding cross-sectional images in the middle (2) and on the right (3). Scale bars, 30 μm.

The three bacteriophages were encapsulated separately into alginate/CaCO_3_ and their properties then examined. The mean size of alginate-encapsulated UAB_Phi20, UAB_Phi78, and UAB_Phi87 as determined by laser diffraction was 124 ± 9 μm, 141 ± 16 μm, and 149 ± 6 μm, respectively. The alginate/CaCO_3_-encapsulated bacteriophages were then characterized by labelling the phage and the alginate with SYBR gold and DAPI, respectively. The 3D spatially superimposed SYBR-gold (green) and DAPI- (blue) fluorescence intensities are shown in Fig. 1. The images show that all of the phages were properly encapsulated in the capsules. This was confirmed by the encapsulation yields of 98.0% ± 1.5 for UAB_Phi20, 99.0% ± 0.1 for UAB_Phi78, and 99.0% ± 0.7 for UAB_Phi87 (Table 1).

The stability of the alginate/CaCO_3_-encapsulated phages was determined by storing freshly encapsulated phages at 4 °C for 6 months and then again measuring the percentage of encapsulated phages. As shown in Table 1, for cold-stored UAB_Phi20 and UAB_Phi78 there was only a slight decrease in the encapsulation percentage compared to the values of freshly encapsulated phages, whereas for cold-stored encapsulated UAB_Phi87 phage there was no loss of encapsulated phage. Similarly, no decrease in the encapsulation percentage was observed after maintaining the alginate/CaCO_3_-encapsulated phages two weeks at room temperature (data not shown).

### Stability of the encapsulated bacteriophages in simulated gastric fluid

**Figure 2:**
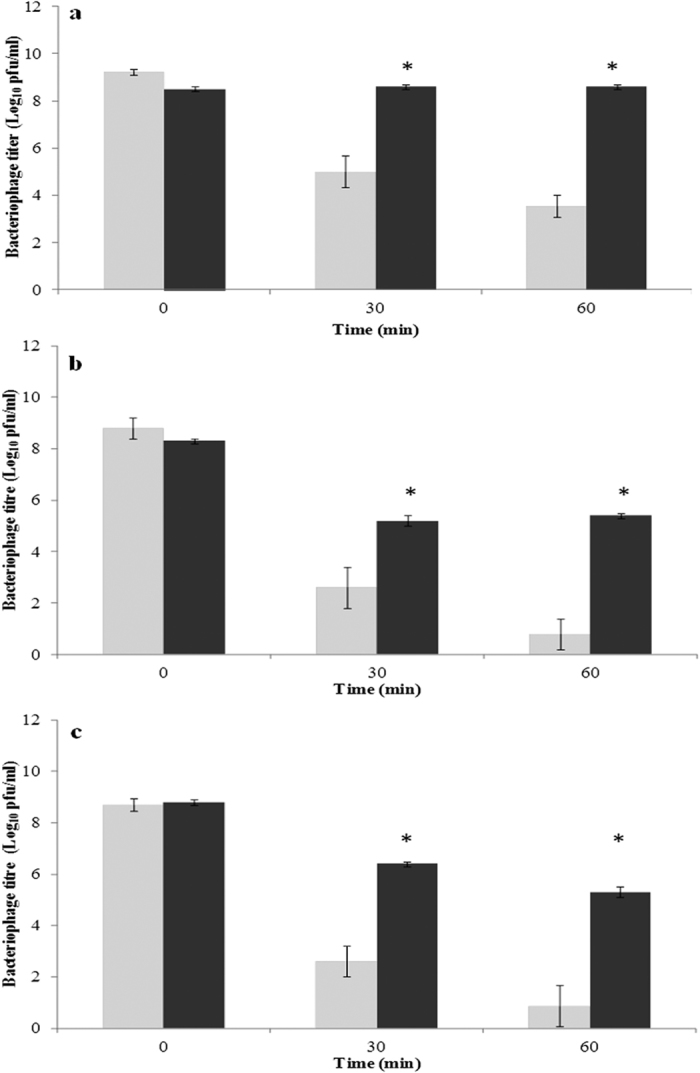
Stability of the non-encapsulated (light gray bars) and alginate/CaCO_3_-encapsulated (dark gray bars) bacteriophages in simulated gastric fluid. (**a**) UAB_Phi20, (**b**) UAB_Phi78, and (**c**) UAB_Phi87. Each value is the average of six independent experiments ± standard deviation. *p < 0.05.

The acid stability of the alginate/CaCO_3_-encapsulated phages was tested by incubating them in simulated gastric fluid (SGF) (pH 2.8) for 60 min. These phages proved to be much more stable under acidic conditions than their non-encapsulated counterparts (Fig. 2) (p < 0.05). Thus, titre losses of phage UAB_Phi78 and phage UAB_Phi87 were 3.1 and 2.4 log10 after 30 min and 2.9 and 3.5 log10 after 60 min of incubation, respectively. Remarkably, there was no reduction in the titre of UAB_Phi20 after 60-min incubation with SGF.

### Encapsulated phage release by simulated intestinal fluid

**Figure 3:**
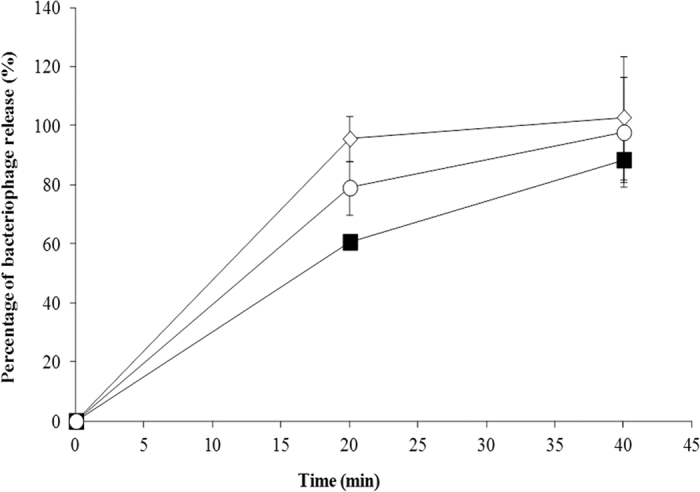
*In vitro* release of alginate/CaCO_3_-encapsulated UAB_Phi20 (○), UAB_Phi78 (▪), and UAB_Phi87 (◊) incubated in simulated intestinal fluid. Each value is the average of three independent experiments ± standard deviation.

The kinetics of phage release from alginate/CaCO_3_ capsules incubated for up to 40 min with simulated intestinal fluid (SIF) was also assessed. After 20 min of incubation in SIF, the percentage of UAB_Phi20, UAB_Phi78, and UAB_Phi87 release was 79.0% ± 9.1, 60.7% ± 2.4, and 95.6% ± 7.6, respectively (Fig. 3). After 40 min, release was nearly complete for all phages (97.7% ± 18.6, 88.4% ± 7.6, and 100.0% ± 20.8, respectively; Fig. 3).

### *In vivo* retention of bacteriophage in the chicken caecum

Previously to do the studies of retention and phage therapy in chicken, we observed that alginate/CaCO_3_ capsules without phages were innocuous in a mouse model (data not shown).

**Figure 4:**
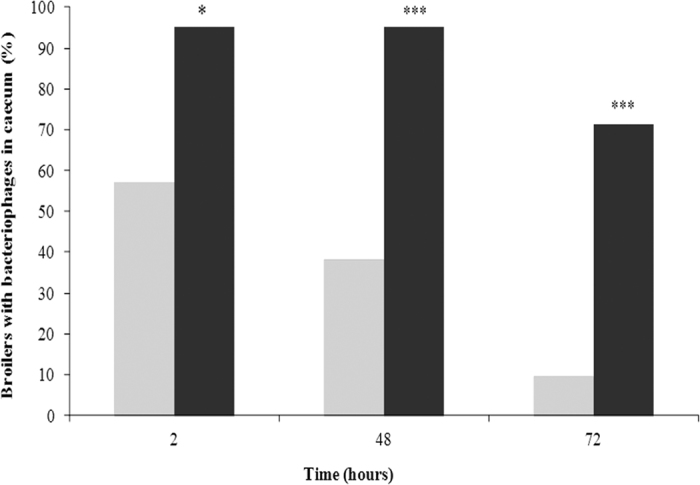
Persistence of the non-encapsulated (light gray) and alginate/CaCO_3_-encapsulated (dark gray) bacteriophages in the broiler caecum. *p < 0.05, ***p < 0.001.

The caecal residence time of a cocktail of the three non-encapsulated and alginate/CaCO_3_-encapsulated phages differed significantly at all three time points tested. Thus, after 2 h the encapsulated phages were retained in 95.2% of the chickens and the non-encapsulated phages in 57.1% (*p* < 0.05; Fig. 4). After 48 and 72 h the differences were still significant (*p* < 0.001): 95.2% vs. 38.1%, and 71.4% vs. 9.5%, respectively (Fig. 4).

### Bacteriophage therapy against *Salmonella* infection in broiler chickens

Cocktails prepared from non-encapsulated and alginate/CaCO_3_-encapsulated phages differed in their long-term efficacies in protecting commercial broiler chickens against *Salmonella* infection, as determined based on the caecal *Salmonella* concentration (Table 2). Thus, within the first 6 days post-infection, reductions in the *Salmonella* concentration in the two treated groups were nearly the same and both were significant compared to the control untreated chickens (*p* < 0.001). However, on day 1 post-infection, a greater reduction of *Salmonella* counts was obtained with the non-encapsulated than the encapsulated phages (*p* < 0.05). The reduction in the *Salmonella* concentration in chickens administered the cocktail of non-encapsulated phages was significant until day 8, the first day after treatment cessation (1.5 log_10_ reduction; *p* < 0.05). By contrast, the protection achieved with the cocktail of alginate/CaCO_3_-encapsulated phages was significant vs. the control from de beginning until day 15 post-infection (days 8 and 10: *p* < 0.001; day 15: *p* < 0.05), with reductions in *Salmonella* counts on days 8, 10, and 15, of 3.6, 3.4, and 1.7 log_10_, respectively. A further comparison of the two phage treatments showed that the reduction achieved with the cocktail of alginate/CaCO_3_-encapsulated phages was significantly greater than that obtained with the non-encapsulated phages from day 8 until the end of the experiment (days 8 and 10: *p* > 0.001; day 15: *p* > 0.05; Table 2). It must be mentioned that we did not find any bacteria isolated from phage-treated groups to be resistant against the three phages of the cocktail administered (manuscript in preparation).

**Figure 5:**
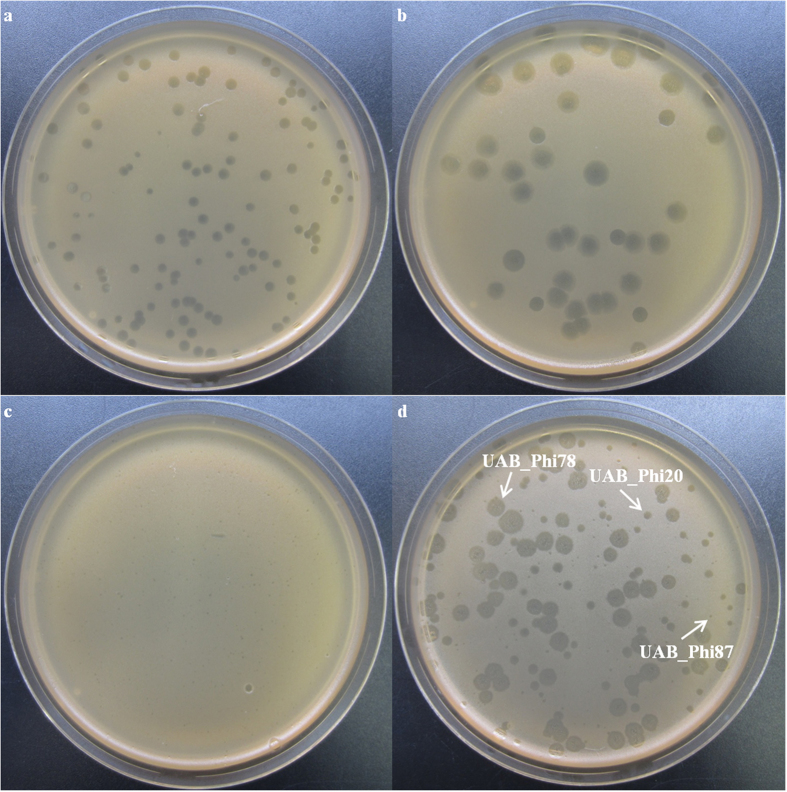
Plaque morphologies of UAB_Phi20 (**a**), UAB_Phi78 (**b**), UAB_Phi87 (**c**), and the cocktail of the three bacteriophages (**d**) in double agar layer plates. Arrows indicate the different plaque morphology of each bacteriophage.

Bacteriophage concentrations remained stable throughout the experiment and were slightly higher in the non-encapsulated (4.2–4.4 log_10_ pfu/g of caecum) than in the alginate-encapsulated (3.8–3.0 log_10_ pfu/g of caecum) group from day 1 to day 10 post-infection (Table 3). However, the concentration in the caecum of the non-encapsulated group was significantly greater (5.1 log_10_ pfu/g; *p* < 0.001) than in the alginate-encapsulated (0.2 log_10_ pfu/g) on day 15. It must be noted that the presence of the three bacteriophages in caecum samples could be demonstrated due to their different plaque morphology (Fig. 5).

## Discussion

Methods allowing the stable and controlled delivery of bacteriophages are of great value in phage therapy. One such method is encapsulation, which in farm animals protects orally administered bacteriophages from the harsh environment of the stomach and facilitates their retention during passage through the intestinal tract to ensure a successful therapeutic effect^24^,^25^,^26^. By maintaining bacteriophage stability, micro- or nano-encapsulation enables not only their oral administration through feed or water but also their administration in other forms, such as inhalation, thereby assuring an adequate dose of the therapeutic phage. Furthermore, encapsulation can overcome other problems related to the application of bacteriophages in food industry processes. The materials used in bacteriophage encapsulation have been examined in several studies^12^,^13^ and include alginate, alone or in combination with other materials^8^,^10^,^16^,^17^,^18^. However, few studies have described the *in vivo* use of alginate-encapsulated bacteriophages^21^,^22^,^27^. Thus, our study is the first to test a cocktail of three alginate/CaCO_3_-encapsulated virulent bacteriophages (UAB_Phi20, UAB_Phi78, and UAB_Phi87)^11^,^23^ as oral therapy in *Salmonella*-infected poultry under farm-like conditions. Phages prepared according to this method were shown to be effective in protecting broilers against infection for up to 15 days.

The encapsulation methodology described herein allows the encapsulation of bacteriophages with different morphologies, without jeopardizing infectivity. The encapsulation efficiency values obtained in this study were ~99%, similar to the percentages reported by other authors^8^,^10^,^16^,^17^,^25^. Moreover, the alginate/CaCO_3_ encapsulated bacteriophages showed excellent stability when stored at 4 °C for 6 months, with minor losses determined only for UAB_Phi20 and UAB_Phi78. Also, the encapsulated phages were stable at room temperature at least for two weeks, period of time that is sufficient for the administration to animals in drinking water.

Another promising feature of the alginate/CaCO_3_ microcapsules was their size (124–149 μm), which was almost ten times smaller than other types of capsules described in the literature^8^,^10^,^16^,^17^,^25^ and facilitated their potential commercial applications. All this is the result of various systematic studies aimed at optimizing the alginate concentration (1.8%) and the posterior curing time of the capsules in the bath of CaCl_2_ (90 min).

Phages orally administered to broilers must withstand the low pH of the stomach contents of the chickens, which is typically in the range of 2.1–3.6^28^,^29^. Under acidic conditions, the alginate/CaCO_3_-encapsulated phages proved to be much more stable than their non-encapsulated counterparts (Fig. 2) (p < 0.05), resulting in titre losses after 60 min of incubation in SGF that were around 5 times lower for phages UAB_Phi78 and UAB_Phi87. There was no loss of encapsulated UAB_Phi20. It is reported that the incorporation of CaCO_3_ slows the gelation rate of alginate capsules, whereas CO_3_^−^ ions dissociated from CaCO_3_ diffuse into the medium and slightly increase the pH as a role of antacid, thus protecting the bacteriophages^10^,^19^. Our results are in accordance with those obtained by^10^, who also reported a good protection effect on the bacteriophage K once encapsulated in a mixture of alginate/CaCO_3_ capsules of a diameter of ~900 μm. However, in our case, this protection effect was achieved even lowering the size of the capsules down to ~150 μm of size.

Another important feature of alginate/CaCO_3_-encapsulated bacteriophages is their release in the animal intestine, where the host pathogen is located. In this study, the three phages exhibited slightly different *in vitro* release kinetics. Thus, whereas almost a complete release of bacteriophage UAB_Phi87 was observed after 20 min of incubation in SIF, UAB_Phi20 and UAB_Phi78 were released more slowly, being this release completed after 40 min (Fig. 3). A potential explanation of the faster release of UAB_Phi87 is that this phage has a larger size than the other two phages, which could provoke the formation of alginate capsules with a lower reticular or cross-linked structure and therefore, a faster release of the encapsulated phages. In addition, a comparison of our release results with those of other authors is difficult, since the size and composition of the microcapsules, the composition of the SIF and the incubation conditions (pH and temperature) differed^8^,^10^,^16^,^18^.

However, bacteriophage release kinetics will undoubtedly differ *in vivo*. Currently, whether phages adhere to the intestinal epithelium^30^ or their presence becomes insignificant in the absence of the bacterial host is unclear^11^,^31^. The mucoadhesive properties of alginate^32^ could prolong the presence and the effect of bacteriophages used in oral phage therapy. Our *in vivo* results of residence time demonstrate that alginate/CaCO_3_ encapsulation enables the significant intestinal retention of the bacteriophages even in the absence of host. Thus, bacteriophages were detected in 71.4% of the chickens 72 h after oral administration of a single dose of the cocktail of encapsulated bacteriophages compared to 9.5% of the chickens treated with the non-encapsulated phages (*p* < 0.001). The percentage obtained with the alginate/CaCO_3_ capsules was better than obtained in a previous *in vivo* study of liposome-encapsulated bacteriophages^24^, perhaps due to the higher encapsulation efficiency achieved with the former.

The cocktail composed of the three alginate/CaCO_3_-encapsulated bacteriophages demonstrated long-term efficacy when used in commercial broilers chickens infected with *Salmonella*, mimicking real farm conditions. Colonization by *Salmonella* was effectively reduced by administering a phage cocktail composed of alginate/CaCO_3_-encapsulated bacteriophages to the poultry 1 day prior to infection with the bacterium and for an additional 7 days during treatment. Moreover, the protective effect was significantly maintained for 1 week after treatment was stopped (on day 15-post infection). Differently, the non-encapsulated bacteriophages quickly loosed their effect once the treatment was stopped. During the first 6 days post-infection, the encapsulated and non-encapsulated cocktails were of similar efficacies, with maximum reductions in *Salmonella* counts of 3.1 and 2.8 log_10_, respectively. Only on the first day of treatment was the non-encapsulate cocktail significantly more effective (reduction of 2.9 vs. 1.3 log_10_; *p* < 0.05; Table 2). This may have reflected the additional time required for the encapsulated phages to accumulate to an effective therapeutic concentration following their release. Then, it is likely that the phage release *in vivo* would be slower than *in vitro* SIF studies. However, the effect of the non-encapsulated cocktail was abolished 1 day after the cessation of treatment (day 7 post-infection) whereas the encapsulated cocktail maintained its effectiveness until the end of the experiment.

This is the first study to report the use of alginate/CaCO_3_-encapsulated bacteriophages as an *in vivo* oral therapy against *Salmonella* infections in poultry. While our results are similar to those achieved with liposome encapsulation^24^, preparation of the alginate/CaCO_3_ capsules is simpler and provides much higher encapsulation rates. Although treatment with the alginate/CaCO_3_ cocktail did not remove *Salmonella* totally, the concentration of *Salmonella* used in the experimental poultry infection was very high (~6 log_10_/g of caecum). Further studies should seek to ascertain the threshold *Salmonella* concentration in poultry farming and if the encapsulated cocktail could eliminate *Salmonella* from the chickens completely in those conditions.

Another important aspect in the success of phage therapy is the relationship between the phages and their bacterial hosts. According to the literature on bacteriophage-bacterial dynamics *in vitro*, the number of phage increases only when the cell density is sufficient, so that the probability of an encounter between the bacteriophages and the bacteria and the subsequent infection of the latter exceeds the probability of phage death^33^. Therefore, the success of phage therapy is largely determined by the relationship between the concentration of the bacteriophages and that of their bacterial host^34^. However, the *in vivo* dynamics are presumably much more complex because multiple external factors (e.g., rapid clearance of the bacteriophages by passive/active host immunity, spatial refuges, and intestinal mucous) influence treatment success^35^.

In our *in vivo* study, the dynamics of the non-encapsulated and encapsulated bacteriophages in controlling *Salmonella* differed which agree with a previous study performed by us with liposome-encapsulated bacteriophages^24^. Thus, with the daily administration of non-encapsulated bacteriophages, their uptake together with the new phage progeny produced in the intestinal tract led to a marked decrease in the *Salmonella* concentration (~50%) 1 day post-infection. Thereafter, until almost day 8 post-infection, the *Salmonella* concentration increased slightly but with significant therapeutic effect. Once the phage uptake was stopped, the equilibrium between phages and bacteria was disrupted and the *Salmonella* concentration increased significantly with respect to day 1 post-infection (days 8 and 10, *p* < 0.05; day 15 post-infection, *p* < 0.001; Table 2). The requirement for continuous administration of bacteriophages along the time to achieve a low population of *Samonella* has also been suggested by other authors^11^,^36^. Several factors as partial emergence of bacteriophage-resistant *Salmonella*, bacterial phenotypic changes, physical refuges or slow growth rate of bacterial cells could explain this fact^35^,^36^. By contrast, when the encapsulated bacteriophage cocktail was administered, the *Salmonella* concentration decreased gradually during all the experiment, regardless of whether treatment was ongoing or had stopped. Therefore, the encapsulation of bacteriophages abolished the need for a continuous treatment to achieve a low bacterial concentration. In this case, the mucoadhesiveness of the capsules and the release kinetics of the bacteriophages from them must be the most important features for this effect. It is remarkable that when the bacteriophage uptake was stopped the bacteriophage concentration remained nearly constant (Table 3) in both treatments, but the *Salmonella* concentration in the gut was higher in the non-encapsulated bacteriophage treatment than in encapsulated one. At this respect, it has been proposed that there is a threshold density of bacteria that must be present in order for the bacteriophage concentration to increase^33^. Further works are needed to identify the *in vivo* mechanism(s) underlying this fact.

In summary, this study demonstrated the utility of a simple, efficient, and inexpensive encapsulation method that can be used with bacteriophages of different morphologies. The small size of the resulting microcapsules (124–149 μm) enables their use in diverse applications in phage therapy. Moreover, alginate/CaCO_3_ encapsulation confers excellent protection against the deleterious effects of gastric juice and promotes greater intestinal retention of the bacteriophages. The results presented here, together with those from our previous investigation of liposome-encapsulated bacteriophages^24^, show that encapsulation is important for a prolonged and successful oral phage therapy in commercial broilers infected with *Salmonella*.

## Methods

### Bacterial strains and growth conditions

Phages UAB_Phi20, UAB_Phi78, and UAB_Phi87 were previously obtained by us from chicken and pig farms^11^,^23^. All three bacteriophages belong to the *Caudovirales* order. UAB_Phi20 and UAB_Phi78 bacteriophages are members of the *Podoviridae* family with icosahedral heads (60 ± 1.5 nm and 66 ± 1.7, respectively) and short tails (13–14 nm) whereas UAB_Phi87 has also an icosahedral head (68 ± 2.7 nm) but a long contractile tail (114 ± 4.3 nm) and derives from the *Myoviridae* family^11^. The genomic features of the three bacteriophages have been recently published^37^. The bacteriophages were propagated in *Salmonella* Typhimurium LB5000 (SGSC181; University of Calgary). Broiler chickens were colonized with *S*. Typhimurium ATCC 14028 Rif^R^. All bacterial strains were grown in Luria-Bertani (LB) broth or on LB agar plates for 18 h at 37 °C. *S*. Typhimurium ATCC 14028 Rif^R^ viable counts were obtained after incubating the bacterium, plated on xylose-lysine-deoxycholate (XLD) plates (Laboratorios Conda, Spain) supplemented with rifampin (75 μg/ml), at 37 °C for 18 h.

### *In vitro* multiplication of bacteriophages

High-titer (1 × 10^11^–1 × 10^12^ pfu/ml in 10 mM MgSO_4_) lysates were obtained from bacteriophages propagated in *S.* Typhimurium strain LB5000 (SGSC181; University of Calgary) and subjected to ultracentrifugation at 51,000 × g for 2 h (Optima^TM^ L-80; Beckman, CA, USA), as previously described^24^. The phage titre was determined by plating serial dilutions (1:10) onto LB plates using the double agar layer method^38^.

### Bacteriophage encapsulation with alginate/CaCO_3_

Encapsulation of the phages using alginate/CaCO_3_ was carried out followed a previously described protocol with slight modifications^10^. Briefly, 500 mg of CaCO_3_ (1%) and 900 mg of alginate (1.8%) were added to 50 ml of each bacteriophage suspension in 10 mM MgSO_4_ at a concentration of 1 × 10^11^ pfu/ml. This mixture was stirred overnight to allow its proper homogenization and then pumped at a rate of 1.5 ml/min into a bath containing 150 ml of 1.8% CaCl_2_ using the ViscoMist^TM^ Air AtomiZing spray nozzle (inner diameter: 381 μm; Lechler Inc., St. Charles, IL) under a continuous flow of nitrogen at a pressure of 3 bars. The gelled capsules were left to harden in the bath for 90 min with slow stirring and then centrifuged at 469 × g for 5 min. The resulting pellet was washed thrice with 10 mM MgSO_4_ and resuspended in 10 mM MgSO_4_ at a final volume of 50 ml.

Particle size distributions were determined by granulometric assays based on laser diffraction (Mastersizer 2000, Malvern Instruments, UK), which was appropriate because of the micrometric size of the encapsulated phages. Laser diffraction is a well-established technique to determine particle size distributions covered by ISO13320 (2009)^39^, which measures the angular variation in intensity of light scattered as a laser beam passes through a dispersed particulate sample. The angular scattering intensity data is then analyzed to calculate the size of the particles responsible for creating the scattering pattern, using the Mie theory of light scattering. The mean particle size is reported as a volume equivalent sphere diameter and expressed in terms of the “volume weighted mean”, as the 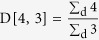, where the diameter (d) has d4 dependence and the number of particles is not inherent in the formulae. The D[4, 3] value was calculated using an algorithm from the Mastersizer Software. The undiluted capsules were measured directly after encapsulation and the D[4, 3] value is the media of three different measurements.

The encapsulation efficiency (EE) of the phages was calculated as EE (%) = 100 − (C_free_/C_total_) × 100, where C_total_ is the concentration of all bacteriophages and C_free_ the concentration of free phages^24^. To determine C_total_, the product of the phage encapsulation was titrated directly. Because divalent ions are essential for the stability of the alginate capsules, degradation of the plated capsules may occur due to the sequestration of divalent ions during gelling of the double agar layer. To quantify C_free_, 0.5 ml of the encapsulation product was filtered through a 0.22-μm PES filter syringe to retain the encapsulated phages. The eluted volume was then titered. The stability of the alginate-encapsulated phages at 4 °C was tested for 6 months using the above-described method for EE determination.

### Microscopy

The phages were labeled with SYBR gold (Molecular Probes, OR, USA) as previously described^24^, and encapsulated as described above, but with the addition of 4′,6-diamidino-2′-phenylindole dihydrochloride (DAPI)-labeled alginate to the mixture to a final volume of 10%. Alginate labeling was performed as previously described, but using DAPI rather than fluoresceinamine^40^. Samples of 30 μl were observed using a Leica TCS SP5 confocal microscope (Leica Microsystems, Germany) but without the resonance scanning mode, as the capsules were large enough to reduce Brownian motion.

### Bacteriophage stability in simulated gastric fluid

The stability of the encapsulated phages in SGF (pH 2.8), consisting of 3 mg pepsin (Sigma-Aldrich, MO, USA)/ml in 0.85% NaCl, was tested as previously described^24^. However, in this study, the solution exhibited a buffering effect till a pH value of 3.8. The bacteriophage titer was determined as described above using aliquots taken at 0, 30, and 60 min of incubation in SGF.

### Alginate/CaCO_3_-encapsulated phage release in simulated intestinal fluid

Phage release from alginate capsules in SIF (pH 8.0), consisting of 1 mg pancreatin (Sigma-Aldrich, MO, USA)/ml, 10 mM bile salts, and 0.85% NaCl^41^, was determined. Thus, a suspension of alginate-encapsulated phages at a concentration of 1 × 10^9^ pfu/ml was inoculated in SIF and incubated in a water bath at 42 °C with agitation to emulate the conditions of the avian intestine. C_total_ and C_free_ were calculated as described above.

### *In vivo* assays in broilers

Both the residence time in the chicken intestine and the effectiveness of a cocktail of alginate-encapsulated or non-encapsulated phages against *Salmonella* was evaluated *in vivo* in commercial broilers (*Gallus gallus*, Ross strain 308; Terra-Avant S.A., Girona, Spain), using an experimental model that mimics farm conditions, as described in a previous study^24^. All *in vivo* animal experiments were performed in strict compliance with the protocols approved by the Comissió d’Ètica en l’Experimentació Animal i Humana (CEEAH) of the Universitat Autònoma de Barcelona (UAB). The approved study was assigned with the authorization number 1953.

Caecum samples were collected from two euthanized chickens and tested using previously described enrichment protocols^24^, to confirm that they were free of *Salmonella* and phages.

A curved oral-dosing needle was used to orally administer the broilers 100 μl of a cocktail containing either the alginate-encapsulated or non-encapsulated phages^24^. The cocktail consisted of a 1:1:1 mixture of the three phages (UAB_Phi20, UAB_Phi78, and UAB_Phi87) at a concentration of 10^11^ pfu/ml in MgSO_4_ buffer without antacid.

### Bacteriophage retention in the chicken caecum

The intestinal residence time of the cocktail of either the encapsulated or non-encapsulated phages was determined over 72 h. Two independent experiments were conducted with two groups of 63 1-day-old chickens. The phage cocktail was administered at a dose of 10^10^ pfu/animal as described previously^24^. After 2, 48, and 72 h, caecum samples were collected from 21 euthanized animals and processed as previously described^24^. The total phage number in the homogenized caecum samples was determined as described above. When direct detection of the phages was not possible, an enrichment procedure was carried out using a previously described method^24^.

### Bacteriophage therapy

Bacteriophage therapy against *Salmonella* was evaluated over 17 days in chickens orally administered the different phage cocktails, as described in a previous work^24^. Briefly, the animals were orally infected on day 0 with a suspension of *S*. Typhimurium ATCC14028 Rif^R^ (10^7^ cfu/animal). Three sequential experiments with groups of 84 commercial broilers (*Gallus gallus*, Ross strain 308) each were conducted. Group 1 corresponded to the *Salmonella* colonization control. Groups 2 and 3 received the non-encapsulated and alginate-encapsulated phages, respectively. In all cases, the oral dose of the three-phage cocktail (10^10^ pfu/animal) was administered once daily for 9 days (from day −1 to day 7 after *Salmonella* infection). The control group was orally inoculated with MgSO_4_ (10 mM). To quantify the *Salmonella* and the phages, 14 chickens per group were euthanized on days 1, 3, 6, 8, 10, and 15 post-infection. Sample processing and determination of the *Salmonella* concentration in caecum samples were done as previously described^24^. The total concentration of bacteriophages was determined as described above. The reduction in the number of bacteria in each treatment was calculated by subtracting the mean caecal concentration (expressed in log 10 units) in groups 2 and 3 from the mean value of the control (group 1).

### Statistical analysis

All results were analyzed using IBM SPSS software. For normally distributed samples, an analysis of variance (ANOVA) and Student’s t test were applied; in cases of a non-normal distribution, the Kruskal-Wallis and Mann-Whitney tests were used.

## Additional Information

**How to cite this article:** Colom, J. *et al*. Microencapsulation with alginate/CaCO_3_: A strategy for improved phage therapy. *Sci. Rep.* **7**, 41441; doi: 10.1038/srep41441 (2017).

**Publisher's note:** Springer Nature remains neutral with regard to jurisdictional claims in published maps and institutional affiliations.

## Figures and Tables

**Table 1:** Percentage (%) of alginate/CaCO_3_-encapsulated phages after their storage at 4 °C for 6 months.

Bacteriophage	Fresh^a^	Stored at 4 °C^a^	
		Three months	Six months
UAB_Phi20	98.0 ± 1.5	84.0 ± 0.7	74.0 ± 8.6
UAB_Phi78	99.0 ± 0.1	89.0 ± 2.3	73.0 ± 4.2
UAB_Phi87	99.0 ± 0.7	92.0 ± 5.9	94.0 ± 5.2

^a^Each value represents the average from three independent experiments ± standard deviation.

**Table 2:** *Salmonella* concentration in the caeca of broilers treated with alginate/CaCO_3_-encapsulated and non-encapsulated bacteriophages.

Day post-infection	*Salmonella* concentration in caecum (log_10_ cfu/g)^a^		
	Control group	Encapsulated group	Non-encapsulated group
1	5.8 ± 0.7	4.5 ± 1.4^b^	2.9 ± 2.3b,^c^
3	6.6 ± 0.5	4.0 ± 1.5^b^	3.3 ± 2.7^b^
6	6.9 ± 0.8	3.8 ± 2.2^b^	4.1 ± 2.1^b^
8	6.7 ± 0.5	3.1 ± 2.5b,^c^	5.2 ± 2.2b,^d^
10	6.4 ± 1.0	3.0 ± 0.9b,^c^	5.7 ± 1.9^d^
15	5.2 ± 1.3	3.5 ± 2.1b,^c^	6.3 ± 1.0^d^

^a^Each value is the average from 14 caecum samples ± standard deviation.

^b^Statistical significance between the control and each treated group (*p* < 0.001 at days 1 to 6; for *p* < 0.001 at days 8 and 10 for the encapsulated group, and *p* < 0.05 at day 8 for non-encapsulated group and at day 15 for encapsulated group).

^c^Statistical significance between the two treated groups *p* < 0.05 at days 1 and 8, and *p* < 0.001 on days 10 and 15.

^d^Statistical significance of the increase of *Salmonella* concentration at days 8 and 10 (*p* < 0.05), and on day 15 (*p* < 0.001), with respect day 1 post-infection.

**Table 3:** Bacteriophage concentrations in the caeca of broilers treated with alginate/CaCO_3_-encapsulated and non-encapsulated bacteriophages.

Days post-infection	Bacteriophage concentration in caecum (log_10_ pfu/g)^a^	
	Encapsulated group	Non-encapsulated group
1	3.8 ± 1.0	4.2 ± 0.6
3	3.5 ± 1.0	4.7 ± 1.7
6	3.6 ± 1.4	4.0 ± 0.8
8	3.8 ± 1.2	4.2 ± 2.1
10	3.0 ± 1.7	4.4 ± 2.7
15	0.2 ± 0.6	5.1 ± 2.2^b^

^a^Each value is the average from 14 caecum samples ± standard deviation.

^b^Statistical significance between the encapsulated and non-encapsulated groups (*p* < 0.001).
